# Efficiency of Several Cytochrome P450 Biomarkers in Highlighting the Exposure of *Daphnia magna* to an Organophosphate Pesticide

**DOI:** 10.3390/toxics10080482

**Published:** 2022-08-17

**Authors:** Elodie Melo de Almeida, Floriane Tisserand, Micaela Faria, Nathalie Chèvre

**Affiliations:** 1 IDYST, Faculty of Geosciences and Environments, University of Lausanne, 1015 Lausanne, Switzerland; 2 School of Biology, Faculty of Biology and Medicine, University of Lausanne, 1015 Lausanne, Switzerland

**Keywords:** xenobiotic detoxification, cytochrome P450, biomarker, *Daphnia magna*

## Abstract

The cytochromes P450 (CYP450) represent a major enzyme family operating mostly in the first step of xenobiotic detoxification in aquatic organisms. The ability to measure these CYP450 enzymes’ activities provides a crucial tool to understand organisms’ response to chemical stressors. However, research on CYP450 activity measurement is still limited and has had variable success. In the present study, we optimize, compile, and compare existing scientific information and techniques for a series of CYP450 biomarkers (EROD, MROD, ECOD, APND, and ERND) used on *Daphnia magna*. Additionally, we explored these CYP450 biomarkers’ activities through the first 5 days of life of daphnids, providing a link between their age and sensitivity to chemicals. In the experiment, daphnids were exposed to an organophosphate pesticide (diazinon) from birth to measure the molecular response of the detoxification process. Our results suggest EROD as the most applicable biomarker for organisms such as *D. magna*, with a higher organophosphate detoxification rate in daphnids that are 2 and 5 days old. Additionally, a larger body size allowed a more accurate EROD measurement; hence, we emphasize the use of 5-day-old daphnids when analyzing their detoxification response.

## 1. Introduction

Freshwater habitats are among the most threatened environments, primarily due to anthropogenic stressors such as increased overexploitation and extended watersheds [1]. In addition to these stressors, a wide range of contaminants resulting from human activities have been released into aquatic environments in recent decades. Chemicals represent one of these stressors. Notorious pesticides employed in agriculture and household practices to control pests, weeds, and plant diseases release many toxic elements, reaching and threatening different environmental compartments, including aquatic environments.

Organisms living in these degraded habitats develop sublethal changes in biochemical endpoints. These endpoints can be used to assess whether exposure causes biological responses in organisms [2]. Cytochromes P450 (CYP450) represent a superfamily of enzymes operating in the first step of the detoxification process and are capable of catalyzing the oxidative biotransformation of most xenobiotic substances [3,4]. As a result of its role in detoxification, CYP450′s activities have been used as biomarkers of chemical exposure; their activation or inhibition is a common response to a range of synthetic chemicals [5]. Furthermore, their gene overexpression has been attributed to pesticide resistance in several insects [6,7]. Hence, the use of biochemical biomarkers may allow early interventions and the protection of wild populations exposed to chemical agents [8]. The ability to measure CYP450 activity is crucial for the comprehension of chemical effects and interactions in species exposed to environmental toxicants.

CYP450 mechanisms have been studied in several species, including humans [9], bacteria [10], plants [11], fish [12], reptiles [2,13], insects [14], and aquatic invertebrates [15,16]. However, to the best of our knowledge, the characterization and the comparison of CYP450 activity measurements in small aquatic invertebrates exposed to xenobiotics are still lacking. The ability to measure CYP450 activities in these species remains aleatory, with the use of different CYP450 biomarkers. As mentioned above, CYP450 represents a superfamily of enzymes whose distribution and activities are differently expressed depending on the species [17,18]. For example, CYP-mediated substrates belonging to the CYP1A subfamily are known to be specific biomarkers of exposure to polychlorinated biphenyls (PCBs) [19] in fish, whereas in seals, the CYP3A family showed more specificity in a case of PCB exposure [20]. Thus, the use of a similar CYP450 biomarker on different species can reflect heterogenous results, leading to misinterpretation [15,18,21,22,23,24] and precluding potential comparison between studies. To address this concern, we considered optimizing and adapting different CYP450 biomarker methods for aquatic macroinvertebrates species in the case of pesticide exposure. Applying *Daphnia magna* as the model organism, we selected five commonly used CYP450 biomarkers that highlight different CYP450 families and examined their activities in a case of organophosphate pesticide exposure. These selected CYP450 biomarkers, namely, ethoxyresorufin-O-deethylase (EROD), methoxyresorufin-O-demethylase (MROD), ethoxycoumarin-O-deethylase (ECOD), aminopyrine-N-demethylase (APND), and erythromycin-N-demethylase (ERND), were chosen in order to investigate a larger type of CYP450 families; EROD and MROD are both related to one CYP450 family, the CYP1A clan [4,16], while ECOD is related to a wider range of families (CYP1, CYP2, and CYP3) [25]. Finally, APND and ERND are related to CYP2B [26] and CYP3A, respectively [23]. Moreover, we optimized and adapted protocols to obtain a similar methodology and to be able to compare the results. The methodology consisted of catalysis driven by CYP450 enzymes of the substrate to a product measurable by fluorescence or absorbance, allowing its quantification. Additionally, we noted that previous studies tested the CYP450 activities of *D. magna* after different times of xenobiotic exposure and at different ages [21,24]. However, sensitivity and response to chemicals fluctuate through age [27,28]. Hence, we also investigated the sensitivity of three selected CYP450 biomarkers in the first 5 days of *D. magna* neonates.

*D. magna* is a zooplankton widely used in environmental toxicological analyzes because of its sensitivity to a wide range of toxic substances [29]. *D. magna* represents a keystone species in the pelagic zone of most freshwater habitats [30], providing a link between primary and higher trophic levels [31]. Hence, *D. magna* is an ideal system for studying the effect of multiple stressors and has been used as an indicator to assess the toxicity due to synthetic detergents because of its short generation time [32], well-studied ecology and evolutionary history, wide geographical distribution, and high sensitivity to changes in environmental conditions. Using *D. magna* as experimental organisms to measure CYP450 biomarkers’ activities brought new challenges. Firstly, substrates commonly used for vertebrates’ organisms might be unsuitable for invertebrates due to differences in CYP450 isoforms [33]. Secondly, the measurement of CYP450 concentrated into target organs such as the liver, hepatopancreas, or gut system are commonly used for larger organisms but not feasible for organisms as daphnids, where the small body size necessitates using the individual as a whole. Finally, aquatic invertebrates have very low CYP450 activities compared to aquatic vertebrates [34].

Organophosphates are among the most frequently used pesticides worldwide [35,36]. In general, organophosphate residues tend to persist for extended periods in water, posing important threats to non-target organisms such as cladocerans and impacting the balance of biological communities [37]. Organophosphate pesticides are known to inhibit CYP450 enzymes. Indeed, the detoxification mechanism of action activated by this type of pesticide involves a release of sulfur molecules during its activation, which form an oxon analogue to the CYP450 enzyme, leading to the inhibition of CYP450 and thus the biomarker [38,39]. Even if this mechanism was demonstrated in fish, it is assumed to be similar in daphnids [35]. Hence, we decided to use an organophosphate pesticide, the diazinon, as a model compound in the current study because of its known mechanism of action. The use of a model compound as the diazinon could allow a better understanding of other organophosphate pesticides and their mechanisms.

In this study, we firstly investigated the signal quality of five CYP450 biomarkers (EROD, MROD, ECOD, APND, and ERND) in *D. magna* exposed to an organophosphate pesticide, the diazinon. Then, we interrogated *D. magna* neonates’ age-specific differences in CYP450 sensitivity using the most relevant biomarkers. As mentioned above, these CYP450 biomarkers are used in ecotoxicology studies, and an accurate characterization of their use is essential and can be relevant in field studies in the model organism being studied.

## 2. Materials and Methods

### 2.1. D. magna Culture Conditions

*D. magna* (Straus, 1820; Cladocera, Daphniidae), originally obtained from the Swiss Federal Institute of Aquatic Science and Technology (Eawag), Zürich, were cultured following the “OECD guideline for Testing of Chemicals” [38]. They were grown in culture with M4 medium (details available in Appendix A, “List of chemicals”) fed with 1.28 × 10^7^ cell/mL/daphnids unicellular algae (Pseudokirchneriella subcapitata), with a light:dark cycle of 16:8 (approximately 800 lux) and a temperature of 21 °C ± 1. The culture medium was refreshed every two days. We used daphnids of 1 to 5 days old for reasons of feasibility, reproducibility, consistency with previous research, and to avoid variability among organisms’ size (daphnids of more than 5-days-old may be carrying eggs).

### 2.2. Chemical Exposure

Diazinon used in experiments was 98.2% pure according to the Certificate of Analysis (Sigma-Aldrich, St. Louis, MO, USA). The stock solutions were prepared in M4 medium at a concentration of 20 mg/L before diluting to 1 µg/L. This solution was analyzed by LC–MS/MS performed by EPFL-ENAC-IIE-GR/CEL, Lausanne, Switzerland, to determine the actual concentration, which was 0.9 µg/L. Based on the EC_50_ (i.e., half maximal effective concentration) of diazinon at 0.89 µg/L determined by Sánchez et al., 1999 [37], the exposure concentrations from the 0.9 µg/L solution were chosen as follows: 0.025, 0.05, 0.1, 0.2, 0.35, 0.5, 0.75, 1, and 1.5 ng/L (nominal concentrations). Moreover, the range of concentrations was selected to gather several daphnids responses (survival, size, and fecundity) [37].

### 2.3. Study Design

#### 2.3.1. *D. magna* Exposed to Several Diazinon Concentrations

Neonates less than 24 h old were taken randomly in pools of twenty daphnids and placed in 250 mL beakers containing 200 mL of the different concentrations of diazinon (0, 0.025, 0.05, 0.1, 0.2, 0.35, 0.5, 0.75, 1, and 1.5 ng/L, nominal concentrations). The exposure time was 5 days. For each concentration, eight replicates of twenty individuals were made and maintained under the same conditions as above. The number of individuals pooled per replicate was determined according to the enzymatic activities’ detection sensibility, tested on previous and complementary work (see Supplementary Materials, Figure S1).

**Figure 1:**
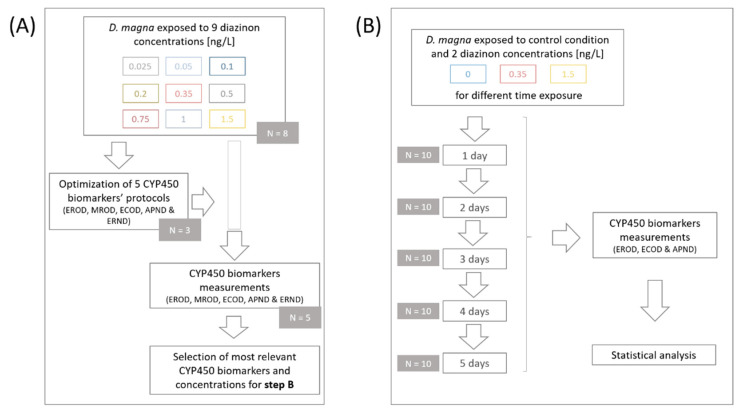
Experimental scheme of the first (**A**) and second (**B**) part of this study. (**A**) After optimization of five different CYP450 biomarkers’ protocols, measurements proceeded on daphnids exposed to nine diazinon concentrations. Most relevant CYP450 biomarkers and concentrations were selected (**A**) to be tested on daphnid neonates exposed for 1 to 5 days (**B**), as well as their basal activity. N corresponds to the number of replicates per condition.

Diazinon solutions and controls were renewed every two days. The 5-day-old daphnids were then removed from their environment and placed in a rinse bath containing only M4 medium to remove any excess chemicals adhered to their body surface, an important precaution to avoid a measurement inhibition [39]. Each pool of twenty individuals was then placed in tubes containing glass beads and set in liquid nitrogen before being stored at −80 °C. Of the eight replicates, three were used to optimize and adapt CYP450 biomarkers protocols for daphnids, and the other five were used to measure CYP450 biomarker (EROD, MROD, ECOD, APND, and ERND) activities in daphnids exposed to nine different diazinon concentrations (Figure 1A).

#### 2.3.2. *D. magna* Exposed to Diazinon during Its First 5 Days of Life

Based on the results obtained in the first part of this study, we decided to expose daphnids to two out of the nine diazinon concentrations, a medium and a high one (0.35 and 1.5 ng/L), to cover a wide range of possible effects induced by diazinon exposure. Additionally, the CYP450 biomarkers showing the most relevant activities in the first part of the design and related to different CYP450 families were selected to be tested in the second part of this study (EROD, ECOD, and APND) (Figure 1B). The exposure time varied from 1 to 5 days and reflected the age of the daphnids exposed. For each concentration and each day, ten replicates of twenty individuals were made and maintained under the same conditions as mentioned above. Again, organism collection was carried out following the above-mentioned protocol; daphnids were first rinsed with M4 medium, placed in tubes containing glass beads, snap-frozen with liquid nitrogen, and stored at −80 °C until biomarker measurements.

### 2.4. EROD, MROD, ECOD, APND, and ERND Activity Measurements

#### 2.4.1. Protein Fraction Preparation

*D. magna* protein extraction to test biochemical biomarkers to assess CYP450 enzymes’ activity consisted of adding 450 µL of crushing buffer in stocked samples placed in the Bertin Cryolis evolutionTM for 1 min (7500 rpm). The crushing buffer was prepared with phosphate buffer (100 mM, pH 7.8) and 0.1% of protease inhibitor (details of protease inhibitor component available in Appendix A, “List of chemicals”). Then, the homogenate was centrifuged at 9000× *g* for 15 min (4 °C). The supernatant was collected to proceed with the protein assay using the QuantiProTM BCA Assay Kit (Sigma-Aldrich) and enzymes’ activity measurements. All manipulations were performed on ice to avoid products’ denaturation.

#### 2.4.2. Protein Concentration

Total protein concentration was measured using the bicinchoninic acid assay (BCA assay). Protein content was quantified using bovine serum albumin (BSA, 0.5 mg/mL) standard dilution series (0–200 µg/mL BSA in phosphate buffer 100 mM, pH 7.8). Diluted samples were incubated with a BCA mix in a transparent 96-well plate for 1 h at 60 °C (350 rpm). Absorbance was read at 562 nm. All assays were performed in triplicate in the following enzyme activity tests, and absorbance or fluorescence was measured using the Tecan Spark^®^ multi-mode microplate reader.

#### 2.4.3. EROD, MROD, and ECOD Activities

A black 96-well fluorometric plate was used to measure EROD, MROD, and ECOD activities. Based on existing methods, protocols of EROD [40,41], MROD [16], and ECOD [21] were optimized. We adapted the existing methods by adjusting the standard range of resorufin and hydroxycoumarin as well as the manual gain setting, which determines the amplification of a detected signal during the fluorescence endpoint measurement.

EROD and MROD activities were determined by measuring the formation of the resorufin and its fluorescence (excitation wavelength of 535 nm and emission wavelength of 590 nm). The assay consisted of the transformation of 7-ethoxyresorufin (EROD) or 7-methoxyresorufin (MROD) into resorufin by CYP450 enzymes present in the microsome samples (i.e., supernatant of the protein fraction extracted from *D. magna*). Regarding ECOD activity, the formation of hydroxycoumarin and its fluorescence was determined as its activity (excitation wavelength of 380 nm and emission wavelength of 480 nm). The ECOD assay consisted of the transformation of 7-ethoxycoumarin into 7-hydroxycoumarin (see Appendix A for EROD, MROD, and ECOD stock solution details and reaction illustrations).

*D. magna* microsome (30 µL) was added with either 7-ethoxyresorufin (concentration in well: 2 µM, EROD), 7-methoxyresorufin (concentration in well: 2 µM, MROD), or 7-ethoxycoumarin (concentration in well: 0.45 µM, ECOD) and NADPH (concentration in well: 0.167 mM) for a final reaction volume of 200 µL. A standard curve of resorufin products (0–0.018 µM in phosphate buffer 100 mM, pH 7.8) was used to convert relative fluorescence units to pM/min/mg for EROD and MROD assays. The standard curve for the ECOD assay was realized using 7-hydroxycoumarin (0–0.075 µM in phosphate buffer 100 mM, pH 7.8) to convert relative fluorescence units to pM/min/mg. NADPH was put just before the microplate reading, because of its fast reactivity on reaction. Fluorescence levels of resorufin and 7-hydroxycoumarin were measured 30 min after adding NADPH to determine the EROD, MROD, and ECOD activities. The manual gain setting was adjusted to 60 on the Tecan Spark^®^ multi-mode microplate reader.

#### 2.4.4. APND and ERND Activities

In a 96-well plate colorimetric assay, APND and ERND activities were measured as described by Peng et al. (2013) [23] and Nash (1953) [42], with some modifications. Their activities were determined by measuring the formation of formaldehyde (see Appendix A for ERND and APND stock solution details and reaction illustrations).

*D. magna* microsome (10 µL) was mixed with NADPH (concentration in well: 83.3 µM) and aminopyrine (concentration in well: 1.16 mM) or erythromycin (concentration in well: 57.6 µM). The plate was incubated for 30 min at 37 °C (350 rpm). The reaction was then stopped by adding Nash solution (0.3 M). Finally, the plate was incubated for 10 min at 60 °C (350 rpm), and absorbance was read at 420 nm. A standard curve of formaldehyde products (0–20 µM in phosphate buffer 100 mM, pH 7.8) was used to convert absorbance units to APND or ERND activities in nM/min/mg.

### 2.5. Statistical Analysis

Data analyses were performed using the software program RStudio (version 4.0.3, R Core Team 2021 [43]). To ensure the normality and homogeneity of variance of our data, we performed the Shapiro–Wilk and Levene tests, respectively. When data were normally distributed, a t-test was performed, and when data were not normally distributed, a Kruskal–Wallis rank-sum test was performed. To detect differences among conditions, a pairwise t-test and a Wilcoxon rank-sum test (with a Bonferroni or a Holm–Bonferroni correction) were performed for parametric and non-parametric data distribution, respectively. The results are expressed as the mean ± standard error. The significance was set at *p* ≤ 0.05.

## 3. Results

### 3.1. Exposure to Diazinon

**Figure 2:**
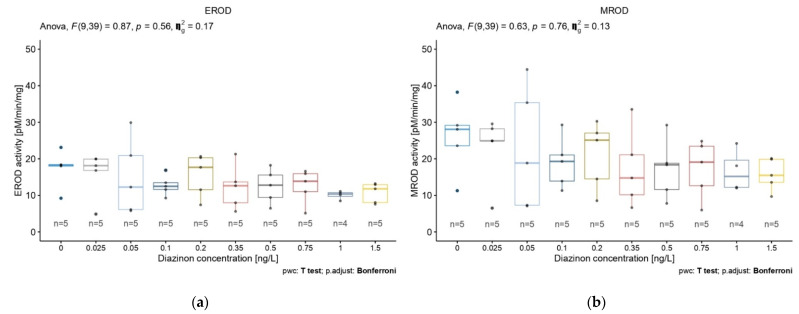

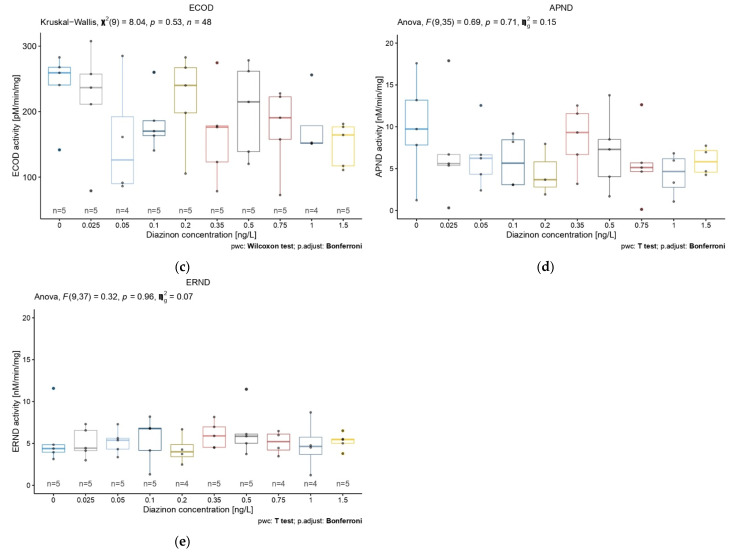
Diazinon effects at nominal concentrations of 0.025, 0.05, 0.1, 0.2 0.35, 0.5 0.75, 1, and 1.5 ng/L on *D. magna* (**a**) EROD, (**b**) MROD, (**c**) ECOD, (**d**) APND, and (**e**) ERND activities after 5 days of exposure. Data are expressed as mean ± SD.

We compared the activity of five biomarkers (EROD, MROD, ECOD, APND, and ERND) in 5-day-old *D. magna* in response to nine concentrations of diazinon. No biomarker activity was significantly affected by increased diazinon concentration (Figure 2). It is noteworthy, however, that the biomarkers EROD, MROD, and ECOD showed similar patterns of activity according to increasing diazinon concentration, with higher concentrations resulting in decreased biomarker activity (Figure 2a–c).

A decrease in APND activity was also observed according to diazinon concentration in two linear patterns (Figure 2d). ERND activity showed no trend according to diazinon concentration (Figure 2e). Based on these results and according to the different biomarkers’ relation to the CYP450 family, we selected EROD, ECOD, and APND as relevant biomarkers in our subsequent study.

### 3.2. Diazinon Effect over Time on EROD, ECOD, and APND Biomarkers

#### 3.2.1. Basal Activity of Biomarkers through the First 5 Days of *D. magna* Life

**Figure 3:**
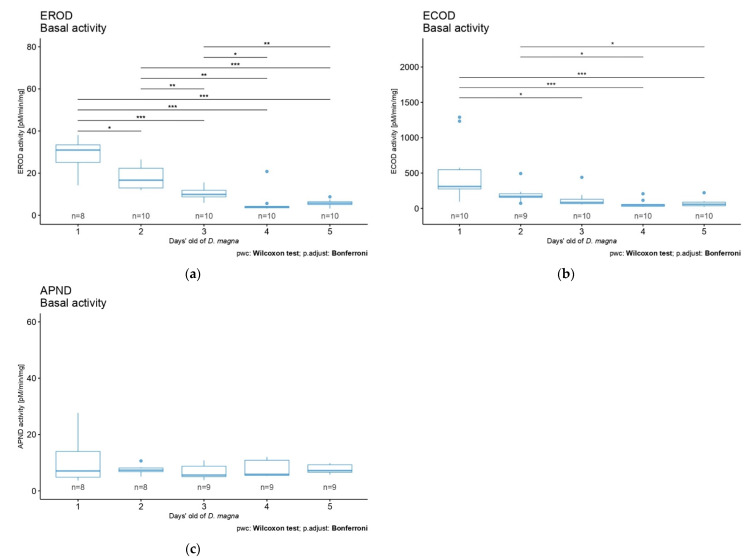
(**a**) EROD, (**b**) ECOD, and (**c**) APND basal activities for the first 5 days in *D. magna*. Significant statistical differences are indicated by * (*p* < 0.05), ** (*p* < 0.01), and *** (*p* < 0.001).

We aimed to measure basal activity of the three target biomarkers (i.e., EROD, ECOD, and APND) through the first 5 days of life of *D. magna* to investigate their activities in non-contaminated conditions. The basal activities values of EROD, ECOD, and APND biomarkers of *D. magna* after 1 to 5 days of life are displayed in Figure 3. Globally, the first day showed the highest activity variability for all three biomarkers. This variability decreased through time, as did the activity values, except for the biomarker APND, for which activity and variability remained constant over the same time period (Figure 3c). Statistical analyses showed a significant difference for EROD and ECOD activities according to exposure time (Figure 3a,b). EROD and ECOD biomarkers basal activities showed significant differences among daphnids of different ages (Kruskal–Wallis rank-sum test, X^2^ = 36.784, df = 4, *p* = 2 ∗ 10^−7^ and X^2^ = 26.88, df = 4, *p* = 2.1 ∗ 10^−5^, respectively). More precisely, EROD activities were significatively different between each day, except between days 4 and 5 (Figure 3a) (pairwise Wilcoxon rank-sum test) (statistical results detail available in Appendix B, Table A1). Concerning ECOD biomarker activities, differences were observed between the following ages: 1–3 days old, 1–4 days old, and 1–5 days, as well as between days 2 and 4 and days 2 and 5 (Figure 3b) (pairwise Wilcoxon rank-sum test) (details on statistical results available in Appendix B, Table A2).

#### 3.2.2. Time Exposure Effect on *D. magna* Exposed to Diazinon

**Figure 4:**
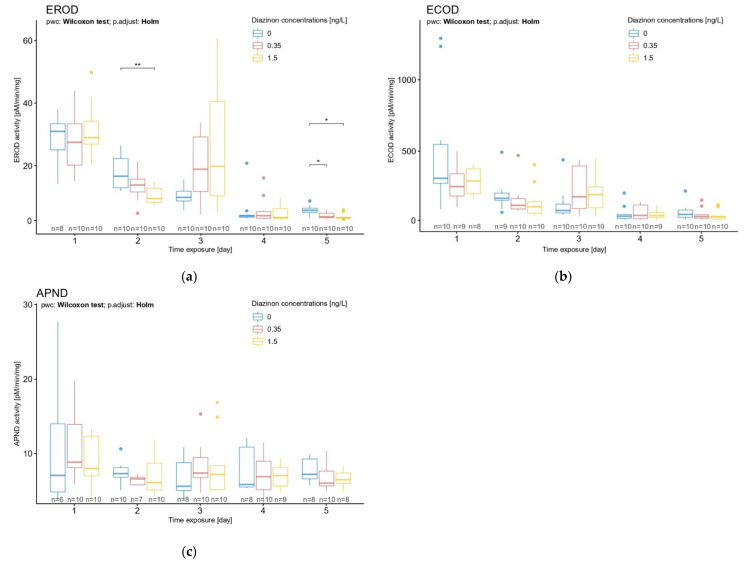
Effects of diazinon at nominal concentrations of 0.35 and 1.5 ng/L according to time exposure (1 to 5 days) on *D. magna* (**a**) EROD, (**b**) ECOD, and (**c**) APND activities. Significant statistical differences are indicated by * (*p* < 0.05) and ** (*p* < 0.01).

We aimed to compare the values of the enzymatic activities in response to three different diazinon concentrations and different exposure times. The effect of pesticide exposure time led to a significant decrease in EROD activity after 2 and 5 days in daphnid neonates (Figure 4a) (Kruskal–Wallis rank-sum test, X^2^ = 8.8335, df = 2, *p* = 0.01 and X^2^ = 10.065, df = 2, *p* = 0.007, respectively). After 2 days of exposure, the effect of the pesticide on EROD was detectable for the diazinon concentration of 1.5 ng/L (Figure 4a) (Wilcoxon rank-sum test, statistic = 88, *p* = 0.006). After 5 days of exposure, the lowest and highest concentrations of diazinon (0.35 ng/L and 1.5 ng/L) showed a significant effect on *D*. *magna* EROD activity (Figure 4a) (pairwise Wilcoxon rank-sum test, statistic = 84, *p* = 0.009 and statistic = 86, *p* = 0.005, statistic = 86, respectively).

No significant diazinon effect was observed on ECOD and APND activities (Figure 4b,c). That said, EROD and ECOD activities showed a similar increase pattern after 3 days of exposure in *D. magna*; a reversed trend was observed in exposed organisms with an increase in activities (Figure 4a,b). Additionally, a decrease in ECOD activity was observed in daphnids after 2 and 5 days of exposure to diazinon, without significant effects (Figure 4b). Note that for all biomarkers, an increase in the activity was observed on day 3, at all concentrations. An increase in response variability was also observed. No explanation was found to this phenomenon. This could be linked to a specific stage of development at day 3, but further research should be performed to clarify this point.

## 4. Discussion

### 4.1. Detoxification Process Detection; CYP450 Biomarkers’ Optimization and Comparison

In the present work, five CYP450 biomarkers used in the literature for organisms as *D. magna* were optimized to develop a similar methodology for the purpose of signal comparison in the case of an organophosphate exposure. We developed protocols to assess in vitro the induction of the biotransformation activities (EROD, MROD, ECOD, APND, and ERND) through the measurement of fluorescence or absorbance metabolites (resorufin, hydroxy-coumarin, and formaldehyde) produced by *D. magna* neonates after 5 days of diazinon exposure.

EROD and MROD biomarker activities showed similar decreasing patterns according to the diazinon concentrations. Both were chosen to verify if we observe similar magnitude of activities as they highlight a shared CYP450 family, the CYP1A [4,16]. Our results of EROD and MROD biomarkers showed comparable signal, attesting their similar CYP450 family target. We therefore chose the most used biomarker in the literature, EROD, during the second part of our research. Moreover, our control sample results showed enzymatic activities with the same order of magnitude as observed in the Peng et al.’s [23] research, with a basal enzymatic activity approximately 15 and 20 pmol/min/mg. Hence, the optimized protocols developed in the present study suggest reproducibility. With EROD being the most common biomarker in the literature, tested on many species and representing a specific biomarker related to one CYP450 family, the comparative study was conducted with only this biomarker. However, despite the sequencing of *D. magna* in 2015 [44,45] and numerous genomic studies [45,46,47], no previous research defined the CYP450 family present in this organism. It is, therefore, interesting to compare biomarkers targeting broader CYP450 families, such as ECOD, and also specialized biomarkers, such as EROD. Indeed, ECOD targets a wider range of CYP450 families (e.g., CYP1, CYP2, and CYP3 [25,48]). Previous studies showed its capacity to detect fungicide exposure effects on *D. magna* [49]. Thus, it was interesting to investigate insecticide effects on ECOD. APND was also selected due to its different measure property (absorbance instead of fluorescence) and its non-specific index reaction to CYP2B and CYP3A families [26,50,51,52]. Finally, ERND was the unique tested biomarker showing no effect of diazinon exposure in *D. magna.* On the contrary, previous work [23,26] observed xenobiotic effects on ERND activities for fish and daphnids exposed to an organochlorine pesticide. We hypothesize that ERND is related to a CYP450 family which is not sufficiently involved in a process of detoxification induced by an organophosphate to observe a diazinon exposure effect, demonstrating that the use of an accurate biomarker according to the type of xenobiotics used is crucial to correctly interpret the detoxification process. Nevertheless, there is no support for this statement found in the literature.

Concerning the different concentrations of diazinon, no significant difference was observed for the biomarkers tested. However, a general decreasing trend according to the concentration of diazinon was observed for all biomarkers, except ERND.

### 4.2. CYP450 Biomarkers’ Basal Activity

**Figure A1:**
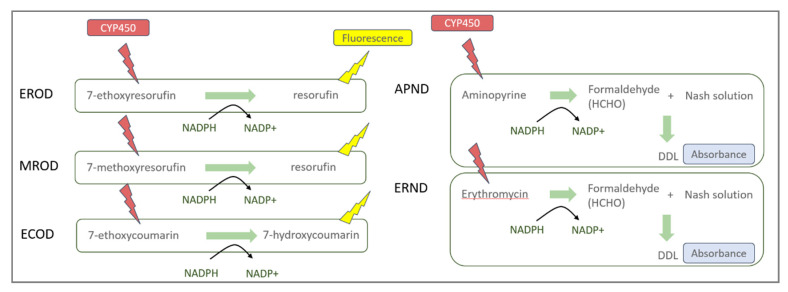
EROD, MROD, ECOD, APND, and ERND biomarker reaction details. For APND and ERND biomarkers, the detection of formaldehyde was based on the absorbance measurement of a colored compound, 3,5-diacetyl-1,4-dihydrolutidine (DDL).

We investigated CYP450 basal activity through the first 5 days of life of *D. magna* using biomarkers selected from the first part of this study: EROD, ECOD, and APND. We reported that EROD and ECOD basal activities decreased through the daphnids’ days of life. Interestingly, previous studies showed similar decreases in basal activity over time [24] in fish [53], gastropod mollusk [15], and *D. magna* [24], but no further analysis was performed on these trends. We believe that in daphnids, a heightened metabolism is required to support several metamorphoses. Indeed, *D. magna’s* juvenile stage is characterized by a high growth rate [54], and its investment in growth could be a hypothetical explanation for the decrease in biomarker activity, leading to a less efficient detoxification process. Moreover, inheritance from mothers can affect offspring’s fitness [55]; maternal effects imply the transfer of material resources to offspring during prenatal development [56,57]. This material transfer from the mother can explain the high CYP450 activity in the very first days of life of daphnid neonates, which then decrease with daphnid age. This inheritance can also explain the high CYP450 activity variability in very young neonates by a potential variable resource transfer from the mother. This can also be explained by the insufficient number of proteins extracted. The small body size of the organism resulted in a poor extraction of proteins, which could lead to a less effective measurement of CYP450 biomarkers. We assume the second exposed hypothesis as the most plausible since the mothers were managed identically, and thus we expected the same material resources transfer to their neonates. These hypotheses, however, remain tentative and require further investigation. CYP450 activities in individuals beyond these first 5 days could be a tentative lead. On the other hand, the use of older organisms would include some beings in reproduction (*D. magna* conditioned in laboratory generally starts reproduction at 6–7 days old). Testing organisms with dissimilar physical states would imply greater variability in results. The APND biomarker showed no activity tendency with the age of daphnids, suggesting absorbance as a less sensitive way of measuring than fluorescence (e.g., EROD and ECOD biomarkers). Indeed, in the case of fluorescence biomarkers, we directly measured the product provided by the substrate cleavage. Regarding the EROD biomarker reaction, the 7-ethoxyresorufin substrate is cleaved by CYP450, providing the resorufin product, which is fluorescent. For the APND biomarker reaction, on the contrary, we indirectly measured the product by adding an acid solution (Nash solution), permitting a quantifiable measurement by absorbance (see Appendix A for CYP450 biomarker reaction details, Figure A1).

### 4.3. Effect of Diazinon through Time Exposure

Here, the most relevant CYP450 biomarkers from previous experiments were selected and their activities were examined in the first 5 days of life of *D. magna* exposed to diazinon. Diazinon exposure significantly affected the enzymatic activity highlighted by EROD after 2 and 5 days of exposure. Despite the lack of significant results, ECOD followed a similar trend, suggesting a lower specificity to the detoxification process occurring in daphnids exposed to an organophosphate. Moreover, we can suppose a smothered effect on the ECOD activity caused by the various CYP450 families highlighted, hiding the activity of the CYP450 family that is characteristically induced during a detoxification process. We know that ECOD highlights a wide spectrum of CYP450 families in humans [25,48]. We can suppose that ECOD may be a useful biomarker when testing species whose relation to CYP450 is poorly studied. Hence, EROD showed specificity as a biomarker in the detoxification process on *D. magna* exposed to an organophosphate pesticide, diazinon. Differences in biomarkers’ responses to pesticide exposure in previous works demonstrate the influence of the pesticide type on the biomarkers’ inhibition or induction. In the present study, the organophosphate inhibited CYP450 activity during the process of detoxification. An accurate understanding of the chemical mechanism of the detoxification process caused by a particular xenobiotic is crucial to correctly interpret CYP450 biomarker activity. The use of another class of pesticide can provide another CYP450 activity response [58], and a lack of consideration could lead to misinterpretation of the results.

## 5. Conclusions

In summary, EROD is the most sensitive biomarker to highlight the effects of diazinon exposure in *D. magna*. Additionally, *D. magna* showed higher sensitivity to diazinon after 2 and 5 days of exposure. We also found that the use of too young daphnids (less than 1 day old) is not advisable because of their variable CYP450 activity. With this study, we attempted to address the challenges and limitations of an in vitro design using *D. magna* and established new protocols adapted to *D. magna* and optimizable for other species of daphnids. We developed an accurate biomarker measurement, adjusted to the type of xenobiotics used and to the age of the daphnids and their sensitivity to chemicals.

## Data Availability

Data are available by contacting the corresponding author.

## Supplementary Materials

The following supporting information can be downloaded at: https://www.mdpi.com/article/10.3390/toxics10080482/s1, Figure S1: EROD activity in healthy *D. magna.* Comparison of EROD activity between samples of pool of 10 and 20 *D. magna* individuals (data ± SD, n = 7). Significant statistical difference is indicated by * (*p* < 0.05).

## Appendix A

### Appendix A.1. List of Chemicals

Bovine serum albumin (BSA, CAS: 9046-46-8, purity: ≥96%), resorufin ethyl ether (7-ethoxyresorufin, C_14_H_11_NO_3_, CAS: 5725-91-7, purity: ≥98%), resorufin methyl ether (7-methoxyresorufin, C_13_H_9_NO_3_, CAS: 5725-89-3, purity: ≥98%), resorufin (C_12_H_7_NO_3_, CAS: 635-78-9, purity: ≥95%), 7-ethoxycoumarin (C_11_H_10_O_3_, CAS: 31005-02-4, purity: ≥99.45%), umbelliferon (7-hydroxycumarin, C_9_H_6_O_3_, CAS: 93-35-6, purity: ≥99%), aminopyrine (4-dimethylaminoantipyrine, C_13_H_17_N_3_O, CAS: 58-15-1, purity: ≥98%), erythromycin (C_37_H_67_NO_13_, CAS: 114-07-8 barium hydroxide (Ba(OH)_2_, CAS: 17194-00-2), formaldehyde solution (CH_2_O, CAS: 50-00-00), acetylacetone (C_5_H_8_O_2_, CAS: 123-54-6), acetic acid (CH_3_COOH, CAS: 1.00062), ammonium acetate (C_2_H_7_NO_2_, CAS: 631-61-8), and diazinon (CAS: 333-41-5, purity: 98.2%) were obtained from Sigma-Aldrich. β-nicotinamide adenine dinucleotide 2′-phosphate reduced tetrasodium salt hydrate (NADPH, CAS:2646-71-1, purity: ≥95%) was bought from Carl Roth, and HaltTM and Protease and Phosphatase Inhibitor Cocktail (100X) (AEBSF ● HCl (100 mM), aprotinin (80 µM), bestatin (5 mM), E-64 (1.5 mM), EDTA (0.5 M), leupeptin (2 mM), and pepstatin A (1 mM)) were purchased from Thermo Fisher Scientific. Alga Pseudokirchneriella Subcapitata were obtained from the Culture Collection of Algae at the University of Göttingen, Germany (SAG). M 4; 1 mL/L of H_3_BO_3_ (57,190 mg/L), MnCl_2_ ● 4 H_2_O (7210 mg/L), LiCl (6120 mg/L), RbCl (1420 mg/L), SrCl_2_ ● 6 H_2_O (3040 mg/L), NaBr (320 mg/L), Na_2_MoO_4_ ● 2 H_2_O (1260 mg/L), CuCl_2_ ● 2 H_2_O (335 mg/L), ZnCl_2_ (260 mg/L), CoCl_2_ ● 6 H_2_O (200 mg/L), KI (65 mg/L), Na_2_SeO_3_ (43.8 mg/L), NH_4_VO_3_ (11.5 mg/L), CaCl_2_ ● 2 H_2_O (293,800 mg/L), and 0.5ml/L of MgSO_4_ ● 7 H_2_O (246,600 mg/L), 0.1 mL/L of KCl (58,000 mg/L), 1 mL/L of NaHCO_3_ (64,800 mg/L), 0.2 mL/L of Na_2_SiO_3_ ● 9 H_2_O (50,000 mg/L), and 0.1 mL/L of NaNO_3_ (2740 mg/L), KH_2_PO_4_ (1430 mg/L), K_2_HPO_4_ (1840 mg/L), and 0.75 mL/day/500 mL of TetraMin [38].

### Appendix A.2. EROD, MROD, and ECOD Stock Solution Details

Stock solutions of 7-ethoxyresorufin (4.145 mM), 7-methoxyresorufin (4.401 mM), 7-ethoxycoumarin (25 mM), and 7-hydroxycoumarin (1.36 mM) were prepared in phosphate buffer (100 mM, pH 7.8), and were stored at −80 °C. Resorufin (0.05 mM) was prepared in dimethyl sulfoxide (DMSO) and was stored at −20 °C.

### Appendix A.3. ERND and APND Stock Solution Details

Stock solutions of aminopyrine (1.160 M), erythromycin (116 mM), and formaldehyde (13.38 M) were prepared in phosphate buffer (100 mM, pH 7.8) and were stored at −80 °C. Nash solution was prepared with acetylacetone (0.02 M), acetic acid (0.05 M), and ammonium acetate (2 M) and was stored in the fridge.

## Appendix B

**Table A1:** Statistical results of EROD basal activity comparison through the 5 first days of life of *D. magna*.

	Pairwise Wilcoxon Rank-Sum Test	
*D. magna* Days Comparison	*p*	Statistic
1 and 2 1 and 3 1 and 4 1 and 5 2 and 3 2 and 4 2 and 5 3 and 4 3 and 5	0.04 0.0009 0.0009 0.0005 0.007 0.005 0.0001 0.02 0.005	71 79 79 80 92 93 100 90 93

**Table A2:** Statistical results of ECOD basal activity comparison through the 5 first days of life of *D. magna*.

	Pairwise Wilcoxon Rank-Sum Test	
*D. magna* Days Comparison	*p*	Statistic
1 and 3 1 and 4 1 and 5 2 and 4 2 and 5	0.02 0.0008 0.0008 0.02 0.04	89 97 97 81 79
