# Supplementary material for: Efficiency of Several Cytochrome P450 Biomarkers in Highlighting the Exposure of Daphnia magna to an Organophosphate Pesticide
Source: Toxics. 2022 Aug 17;10(8):482. doi: 10.3390/toxics10080482 (PMC9416226; doi:10.3390/toxics10080482)
Supplement: Supplementary file 1 [file toxics-10-00482-s001.zip › toxics-1846886-supplementary.pdf]

toxics

MDPI

Supplementary Materials: Method optimization of pollution biomarkers measurements in *Daphnia magna* – pool size effect

Elodie Melo de Almeida, Floriane Tisserand, Nathalie Chèvre

Introduction

Previous studies used different quantities of *D. magna's* individuals [1–3] to test cytochrome P450 biomarkers. Our objective was to test two different small pool size of daphnids, i.e. two different amount of daphnids' individuals per sample, in order to find out the best compromise between feasibility and quality of CYP450 biomarker signal. Pools of 10 and 20 daphnids were tested to improve the detection signal of ethoxyresorufin-O-deethylase (EROD) by fluorescence using the Tecan Spark multi-mode microplate reader. Healthy daphnids were used to proceed the comparison.

Method

Chemical exposure

Same *D. magna* strain, with the same culture conditions, were used as in our study. Five days' old daphnids were used to measure the activity of cytochrome P450 (EROD) using the Tecan Spark® multi-mode microplate reader.

Protein fraction preparation and EROD activity measurement

Protein fraction extraction and EROD activity determination were done similarly as in our study (c.f. 2.4.1 Protein fraction preparation and 2.4.3. EROD, MROD, and ECOD activities, respectively).

Statistical analysis

To compare the variability of EROD activity in pool of 10 and 20 daphnids, seven samples were done for each pool sizes. A Levene's test was used to test the equality of variances between the two sizes pool.

Results

Effect of the pool size

EROD (Levene's test, p-value = 0.01033, F = 9.2229, df = 1) activity in healthy daphnids showed a significant difference in variances between the pool of 10 and 20 daphnids (Figure S1). Data collected for samples with pool of 20 daphnids showed a smaller variance among the 7 samples.

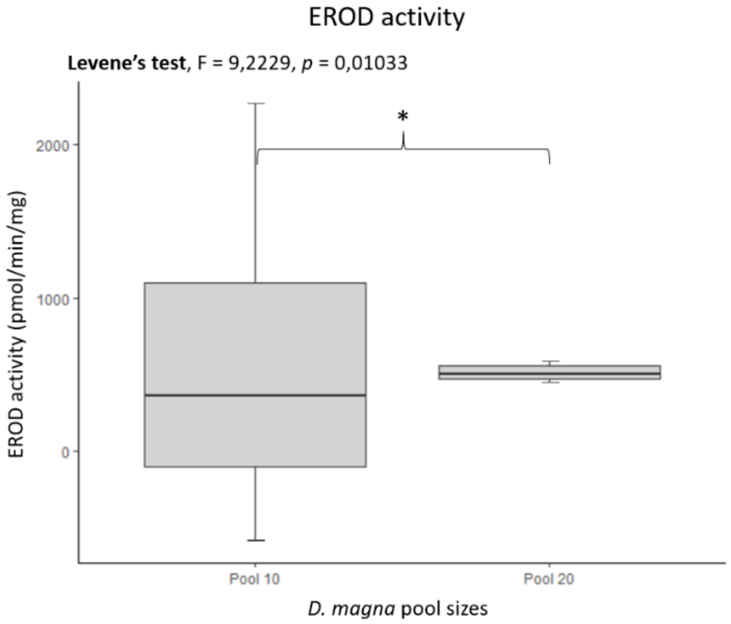

Figure S1. EROD activity in healthy *D. magna*. Comparison of EROD activity between samples of pool of 10 and 20 *D. magna* individuals (data ± SD, n = 7). Significant statistical difference is indicated by * ($p < 0.05$).

References

1. Gottardi, M.; Kretschmann, A.; Cedergreen, N. Measuring Cytochrome P450 Activity in Aquatic Invertebrates: A Critical Evaluation of in Vitro and in Vivo Methods. *Ecotoxicology* **2016**, *25*, 419–430, doi:10.1007/s10646-015-1600-z.
2. Wang, L.; Peng, Y.; Nie, X.; Pan, B.; Ku, P.; Bao, S. Gene Response of CYP360A, CYP314, and GST and Whole-Organism Changes in Daphnia Magna Exposed to Ibuprofen. *Comparative Biochemistry and Physiology Part C: Toxicology & Pharmacology* **2016**, *179*, 49–56, doi:10.1016/j.cbpc.2015.08.010.
3. Peng, Y.; Luo, Y.; Nie, X.-P.; Liao, W.; Yang, Y.-F.; Ying, G.-G. Toxic Effects of Triclosan on the Detoxification System and Breeding of Daphnia Magna. *Ecotoxicology* **2013**, *22*, 1384–1394, doi:10.1007/s10646-013-1124-3.
